# Detailed Versus Simplified Dietary Self-monitoring in a Digital Weight Loss Intervention Among Racial and Ethnic Minority Adults: Fully Remote, Randomized Pilot Study

**DOI:** 10.2196/42191

**Published:** 2022-12-13

**Authors:** Michele L Patel, Angel E Cleare, Carly M Smith, Lisa Goldman Rosas, Abby C King

**Affiliations:** 1 Stanford Prevention Research Center Department of Medicine Stanford University School of Medicine Palo Alto, CA United States; 2 Stanford University Stanford, CA United States; 3 Department of Epidemiology & Population Health Stanford University School of Medicine Stanford, CA United States

**Keywords:** weight loss, obesity, behavioral intervention, self-monitoring, race, ethnicity, digital health, diet tracking, engagement, randomized controlled trial, RCT, mobile phone

## Abstract

**Background:**

Detailed self-monitoring (or *tracking*) of dietary intake is a popular and effective weight loss approach that can be delivered via digital tools, although engagement declines over time. Simplifying the experience of self-monitoring diet may counteract this decline in engagement. Testing these strategies among racial and ethnic minority groups is important as these groups are often disproportionately affected by obesity yet underrepresented in behavioral obesity treatment.

**Objective:**

In this 2-arm pilot study, we aimed to evaluate the feasibility and acceptability of a digital weight loss intervention with either detailed or simplified dietary self-monitoring.

**Methods:**

We recruited racial and ethnic minority adults aged ≥21 years with a BMI of 25 kg/m^2^ to 45 kg/m^2^ and living in the United States. The Pacific time zone was selected for a fully remote study. Participants received a 3-month stand-alone digital weight loss intervention and were randomized 1:1 to either the *detailed* arm that was instructed to self-monitor all foods and drinks consumed each day using the Fitbit mobile app or to the *simplified* arm that was instructed to self-monitor only red zone foods (foods that are highly caloric and of limited nutritional value) each day via a web-based checklist. All participants were instructed to self-monitor both steps and body weight daily. Each week, participants were emailed behavioral lessons, action plans, and personalized feedback. In total, 12 a priori benchmarks were set to establish feasibility, including outcomes related to reach, retention, and self-monitoring engagement (assessed objectively via digital tools). Acceptability was assessed using a questionnaire. Weight change was assessed using scales shipped to the participants’ homes and reported descriptively.

**Results:**

The eligibility screen was completed by 248 individuals, of whom 38 (15.3%) were randomized, 18 to *detailed* and 20 to *simplified*. At baseline, participants had a mean age of 47.4 (SD 14.0) years and BMI of 31.2 (SD 4.8) kg/m^2^. More than half (22/38, 58%) were identified as Hispanic of any race. The study retention rate was 92% (35/38) at 3 months. The *detailed* arm met 9 of 12 feasibility benchmarks, while the *simplified* arm met all 12. Self-monitoring engagement was moderate to high (self-monitoring diet: median of 49% of days for *detailed*, 97% for *simplified*; self-monitoring steps: 99% for *detailed*, 100% for *simplified*; self-monitoring weight: 67% for *detailed*, 80% for *simplified*). Participants in both arms reported high satisfaction, with 89% indicating that they would recommend the intervention. Weight change was −3.4 (95% CI −4.6 to −2.2) kg for *detailed* and −3.3 (95% CI −4.4 to −2.2) kg for *simplified*.

**Conclusions:**

A digital weight loss intervention that incorporated either detailed or simplified dietary self-monitoring was feasible, with high retention and engagement, and acceptable to racial and ethnic minority adults.

**Trial Registration:**

ASPREDICTED #66674; https://aspredicted.org/ka478.pdf

## Introduction

### Background

Obesity is a pervasive health concern in the United States that disproportionately affects many racial and ethnic minority groups [1]. For example, Hispanic adults have an obesity prevalence of 45%, whereas non-Hispanic Black adults have a prevalence of 50% [1]. Excess weight increases the risk of chronic diseases [2] and adverse events from COVID-19 [3]; racial and ethnic minority groups often face a higher risk for these diseases than non-Hispanic White adults or experience greater risk at lower BMI levels [4,5].

Behavioral weight loss interventions are the gold standard for treating obesity [6], with weight loss outcomes of up to 8% by 6 months [7]. These interventions involve diet and exercise goals, frequent counseling, and behavioral strategies. However, few studies have prioritized enrolling racial and ethnic minority groups or creating tailored interventions for these groups [8,9]. The lack of adequate representation of these groups limits generalizability, making it unclear whether the interventions are indeed suitable for populations who might benefit the most from weight loss. Digital health interventions that deliver treatment remotely have the potential to reach broad populations who may otherwise not be able to access behavioral obesity treatment and can minimize barriers to enrollment in these studies. Stand-alone interventions (ie, those without human counseling), in particular, offer greater scalability and can facilitate modest weight loss [10].

A core component of behavioral weight loss interventions is self-monitoring dietary intake [11,12]. The most popular approach, according to recent systematic reviews [13,14], involves detailed self-monitoring of all foods and drinks consumed every day, along with the portion sizes and caloric value of those items. The act of self-monitoring allows individuals to pay attention to their behavior and gain feedback on how specific actions impact their weight. Self-regulation theories, including Social Cognitive Theory, posit that behavior change occurs through the comparison of one’s behavior to one’s goals or past performance [15,16]. Indeed, self-monitoring dietary intake is among the strongest predictors of behavior change [17] and weight loss [18]. Moreover, using digital tools for self-monitoring promotes engagement, compared with paper-based self-monitoring [13]. The advantages of using digital platforms for self-monitoring include immediate personalized feedback, reduced tracking time with automatic calculations of total caloric intake and nutrients, and high portability of mobile health tools, which increases the likelihood of use while reducing retrospective reporting errors.

However, studies that examined the trajectory of dietary self-monitoring engagement have consistently observed prominent declines over the course of an intervention [19-22], which suggests that this detailed dietary self-monitoring approach is often perceived as burdensome. The burden of dietary self-monitoring has been defined by Turner-McGrievy et al [23] as time-intensive and active efforts to initiate, use, and record eating events. As such, simplified or abbreviated approaches to self-monitoring dietary intake are needed to counteract this decline in engagement. These simplified approaches could lower burden by making it easier or quicker to self-monitor one’s diet, which has the potential to sustain engagement levels for longer periods. They could also increase the proportion of people who feel confident self-monitoring by requiring fewer health literacy and numeracy skills. Examples of specific types of simplified strategies include self-monitoring only less routine, less nutritious, or highly caloric foods; these approaches were recommended by experts in behavioral weight management via a Delphi study [24].

Several weight loss studies have tested simplified dietary self-monitoring approaches [23,25-42]. Five of these studies were randomized controlled trials (RCTs) that empirically compared a simplified to detailed self-monitoring approach [25-29]. In all 5 studies, the detailed arms involved daily self-monitoring of all foods consumed along with their caloric intake, whereas the simplified arms varied in their approach, and involved daily self-monitoring of only highly caloric foods via an investigator-designed app [25], photos of foods consumed via the MealLogger commercial app [26], bites of food consumed via a wearable Bite Counter device [27], dietary lapses via an investigator-designed app [28], or 8 weeks of detailed self-monitoring via a paper diary then transitioning to self-monitoring with checklists of estimates of portion size and fat content rather than tracking calories [29].

No studies have tested a simplified versus detailed diet self-monitoring approach among adults from US racial and ethnic minority groups (either 100% of the sample or analyzing outcomes by race or ethnicity). Of the 5 aforementioned RCTs, the proportion of participants from a racial or ethnic minority group ranged from 19% to 21% or was not reported. This is an important gap to address, given the need to develop effective obesity treatments that can be disseminated to racially and ethnically diverse populations. One challenge to detailed dietary self-monitoring is that nutrition databases used in mobile platforms are sometimes missing food items, including foods specific to a geographic region [43] or different types of ethnic foods [44]. As a result, when users have to create many new food entries, self-monitoring becomes more effortful, which could lead to steeper declines in engagement. Understanding both engagement in and the acceptability of detailed and simplified dietary self-monitoring approaches among racial and ethnic minority groups is therefore vital for creating an intervention that meets the needs of its users.

### Objectives

To address this gap, the Spark Pilot Study aimed to evaluate the feasibility and acceptability of a stand-alone, digital behavioral weight loss intervention with either *detailed* or *simplified* dietary self-monitoring in a racially and ethnically diverse sample of US adults. In particular, we examined the reach to our target population, feasibility of our study procedures (eg, study retention) and interventions (eg, engagement in self-monitoring), acceptability of the interventions, and descriptive accounts of our exploratory outcome variables at baseline, 1 month, and 3 months. We conducted a fully remotely delivered trial with all interventions, assessments, and study procedures conducted remotely. This approach allows for greater reach and recruitment speed and may be more acceptable to racial and ethnic minority groups than in-person methods [8,45]. If feasibility and acceptability are established, the next step would be to conduct a fully powered trial to compare the efficacy of a detailed versus simplified dietary self-monitoring strategy among our target population.

## Methods

### Study Design Overview

The Spark Pilot Study was a 2-arm, parallel group, randomized pilot study of a 3-month digital weight loss intervention. Participants were randomized to either the detailed dietary self-monitoring arm (*detailed*) or the simplified dietary self-monitoring arm (*simplified*). We followed the Consolidated Standards of Reporting Trials (CONSORT) guidelines for pilot studies [46]. Given the feasibility and acceptability aims, it was not considered by the funding agency to be a clinical trial; thus, we did not preregister on ClinicalTrials.gov, and instead, we preregistered on AsPredicted, a platform for preregistering research studies (#66674) [47].

### Ethics Approval

All study procedures and human subject research ethics were approved by the Stanford University School of Medicine Institutional Review Board (protocol #59400; approval date: January 27, 2021).

### Participant Compensation

The participants provided written informed consent before enrollment in the study. They were compensated a maximum of US $50 (via e-gift cards) for their completion of assessments, as follows: US $20 at 1 month, US $20 at 3 months, and an additional US $10 for completion of all 4 dietary recalls. The study data are deidentified.

### Participants

Inclusion criteria were adults aged ≥21 years who self-identified as a member of at least one US racial or ethnic minority group; who had a BMI of 25.0 kg/m^2^ to 45.0 kg/m^2^, which corresponds to having overweight or obesity [2]; who owned a smartphone and had access to a personal email account; who were willing to install the Fitbit mobile app on their phone; who were proficient in the English language; who were living in the United States in the Pacific time zone; and who were interested in losing weight through behavioral strategies. The exclusion criteria were concurrent enrollment in another weight management intervention; loss of ≥4.5 kg (ie, 10 lbs) in the past 6 months; current use of a weight loss medication; prior or planned bariatric surgery; current, recent, or planned pregnancy during the study period; currently breastfeeding; living with someone else participating in the study; inability to engage in moderate forms of physical activity akin to brisk walking (assessed via the Physical Activity Readiness Questionnaire for Everyone [PAR-Q+]; [48]); potential contraindications to losing weight due to a serious medical condition (eg, cancer or dementia) or medication; a history of an eating disorder or cardiovascular event or uncontrolled diabetes mellitus that could predispose an individual to be better suited for a more intensive or different type of intervention; and investigator discretion for safety reasons.

### Recruitment

We conducted the study using a fully remote trial design. Enrollment occurred on a rolling basis until our target sample size was met. Participants were recruited from May to June 2021 via free, remote strategies, including ResearchMatch (a web-based US national registry of volunteers interested in participating in research), an institute-specific diabetes registry, and Nextdoor (Nextdoor Holdings, Inc, a web-based neighborhood networking service). Recruitment materials included a brief description of the study and eligibility criteria, along with a link to a web-based screening questionnaire on REDCap (Research Electronic Data Capture; Vanderbilt University), a secure, web-based software platform [49]. Individuals who completed the web-based screen and were eligible and interested were prompted to provide contact information. An automated email was then sent to these individuals to prompt them to sign up for a remote baseline visit via a scheduling website, Calendly, and watch an orientation video. The orientation video was 11 minutes in length and provided an overview of the study objectives, the purpose of randomization, expectations for study participation, and a study timeline. The orientation video was intended to heighten research literacy and trust and increase knowledge of the time commitment and activities involved with study participation to help individuals decide whether the study is a good fit for them and ultimately increase study retention [50,51].

### Study Procedures

#### Baseline Tasks

**Figure 1:**
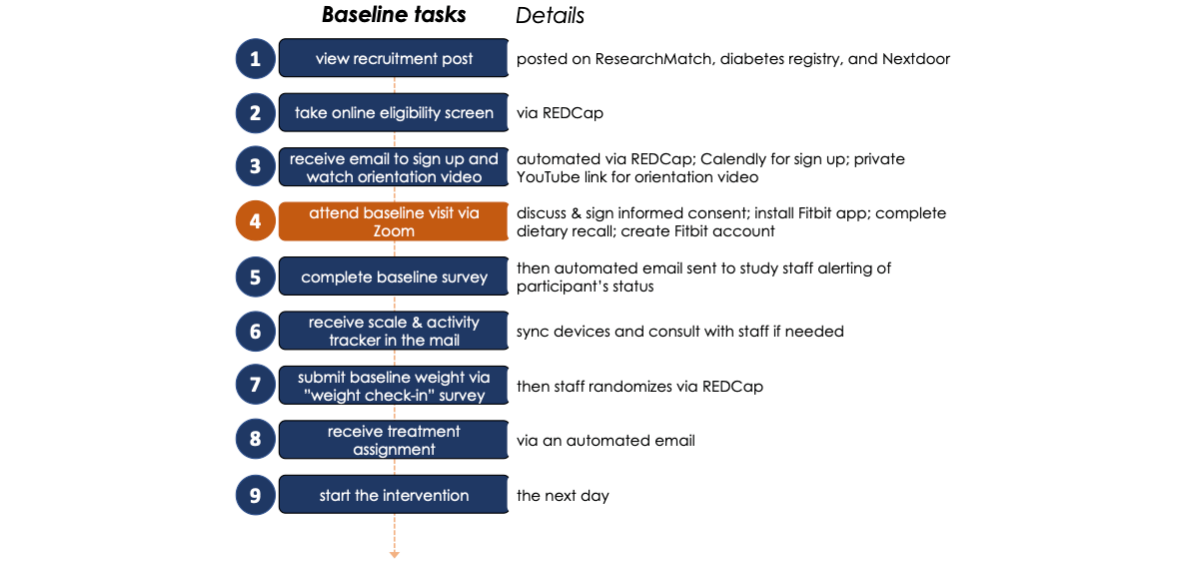
Remote study procedures before starting the intervention from the participant’s perspective. REDCap: Research Electronic Data Capture.

An overview of remote tasks completed before randomization is shown in Figure 1. Briefly, during the 1- to 1.5-hour remote baseline visit held via Zoom videoconference, trained study personnel reviewed the purpose and procedures of the study with participants, assessed the capacity to give consent, and obtained informed consent using REDCap’s electronic signature feature. Participants also completed a dietary recall assessment. The study personnel created a unique Fitbit account for each participant, and participants installed the Fitbit app and set it up with the sections, called *tiles*, they were going to use (eg, keep the *steps* tile and hide the *track water* tile). At the end of the baseline visit, the participants received a link to the baseline survey. Once completed, study personnel shipped to the participants’ homes a Fitbit activity tracker (Inspire 2) and a Withings e-scale (Withing Body) from their respective consumer websites. Once both these devices were received, an email was sent to participants that provided information on syncing their devices and prompted them to weigh themselves the following day using a standardized protocol (see the *Data Collection* section). The principal investigator (MLP) then randomized each participant to a treatment arm using REDCap’s randomization module with stratification for gender (men, women, and other gender). The allocation sequence was generated using Microsoft Excel’s random number generator and stored in REDCap. To note, during the period between enrollment (the baseline visit) and randomization, participants were asked to maintain their current health behaviors. Once randomized, participants received an automated email with their treatment assignment, intervention details, and goal sheet and were instructed to start the intervention the next day. Study personnel were available for troubleshooting via phone or email to assist in syncing devices.

#### Data Collection

Study assessments were conducted remotely at baseline, 1 month, and 3 months. Survey questions were administered in English via REDCap. Data collection ended on October 8, 2021. At each assessment time point, participants received an automated email instructing them to complete a weight check-in survey and a general survey, both administered via REDCap.

Weight was obtained using a commercially available electronic scale (Withings Body), and participants were instructed to follow standardized procedures [52] at the 3 assessments. Specifically, participants were instructed to weigh themselves on the scale in the morning before eating or drinking and after emptying their bladder; place the scale on a hard surface; remove all articles of clothing and accessories; step on the scale and record the value; and repeat it 2 more times for a total of 3 weight measurements. Collecting weight via commercial scales has demonstrated high concordance with weights measured in a clinic setting [53]. Initially, we intended for participants to have their weight automatically synchronize from the scale to the Fitbit app via Wi-Fi or Bluetooth, but the scale we originally intended to use was removed from the commercial store shortly before the study started, and the institutional review board did not approve of the use of the wireless features of the replacement e-scale by the time recruitment ended. Therefore, in accordance with our protocol, we asked participants to manually input the weight value into the weight check-in survey. Reminders to complete these tasks were sent via SMS text message or email. No blinding occurred for treatment assignment, assessment, or analysis, owing to resource constraints. Participants were informed before enrollment about the 2 types of treatments to which they may be assigned and were, by design, not blinded to treatment. The study data were collected and managed using REDCap.

### Intervention

#### Overview

All participants received a 12-week behavioral weight loss intervention. This intervention was a stand-alone treatment, meaning that no human counseling was provided. It included the following empirically supported components: a weight loss goal, 3 domains to self-monitor daily (steps [54,55], body weight [56], and diet [14]) via digital tools [13], weekly tailored feedback on self-monitoring behaviors [57], and weekly behavioral lessons and action plans [17,58,59]. These components are supported by Social Cognitive Theory [16] and are intended to promote behavior change through increased self-regulation, self-efficacy, and outcome expectations.

#### Core Components

##### Fitbit Mobile App

We used a commercially available app from Fitbit that is free on iPhone and Android platforms. The app was set up with the help of study personnel to reflect the weight loss goals and only the self-monitoring components (tracking steps and weight and tracking diet if in the *detailed* arm) to which each participant was assigned (in the form of app tiles). In-app graphical feedback allowed participants to view their self-monitoring progress in real time.

##### Weight Loss Goal

All participants received a goal of 5% weight loss by 3 months. This goal is consistent with obesity treatment guidelines [6] and equates to a weekly weight loss goal of 0.45 kg to 0.91 kg (ie, 1 to 2 lbs) per week, depending on the initial weight.

##### Self-monitoring Steps and Adaptive Step Goal

The participants were instructed to self-monitor their step count daily using a wrist-worn Fitbit activity tracker (Inspire 2). In conjunction with this self-monitoring goal, a daily step goal was given that adapted weekly based on progress. The initial week’s step goal was based on the participant’s baseline scores on the Godin Leisure-Time Exercise Questionnaire (GLTEQ) leisure score index [60,61], with scores ranging from 0 to 13 (interpreted as *insufficiently active*) assigned to a goal of 5000 steps per day (n=10), scores of 14 to 23 (*moderately active*) assigned to 7000 steps per day (n=7), and scores ≥24 (*active*) assigned to 10,000 steps per day (n=21).

Starting in the second week of the intervention, an adaptive step goal was given based on an algorithm adapted from previous studies [62,63], whereby the 60th percentile of the past week’s daily step counts, rounded up to the nearest multiple of 25, was assigned as the subsequent week’s daily step goal. For example, a week with daily steps of 5000, 5100, 6000, 6500, 7000, 8200, and 8500 would result in a step goal of 6800 per day in the subsequent week. The Fitbit activity tracker synced with the Fitbit app to allow participants to view their progress toward the step goal.

##### Self-monitoring Weight

The participants were instructed to self-monitor their body weight daily using a commercially available scale (Withings Body). In a change to our protocol (see *Study Procedures)*, we asked participants to manually input the weight value into the Fitbit app each day. In the app, participants could view their progress toward their weight goal.

##### Tailored Feedback

Each week, participants received an email with tailored, automated feedback pertaining to their progress on their 3 assigned self-monitoring goals (eg, the number of days tracked steps last week), their dietary and step behaviors (eg, “took an average of 9,525 steps per day” last week), and weight change in the past week along with overall weight change since the start of the study. The study team generated feedback by first retrieving data weekly from Fitabase (Small Steps Lab, LLC), a software data management platform. Then, they inputted these data into an Excel spreadsheet that contained all active participants and their goals and semiautomatically generated the progress report using Microsoft Word’s Mail Merge feature. The feasibility of this approach was demonstrated in a prior trial by the investigative team [19].

##### Reminders of Goals

Each week, participants were reminded of their 6 goals (ie, 3 self-monitoring goals along with their personalized weight loss goal, step goal, and either calorie goal or red zone food goal, depending on their treatment assignment). These reminders were attached to the end of each week’s progress report, sent via email.

##### Behavioral Skills Training

Each week, participants received a separate email with theory-informed skills training materials that include structured behavioral lessons on nutrition and physical activity, as well as corresponding action plans. These materials were adapted from gold standard weight loss curricula [64,65]. The lessons included topics such as reading nutrition labels and promoting physical activity (see Textbox 1 for details). Embedded in this email was a link to a brief action plan survey (set up on Qualtrics) that incorporated motivational interviewing [66,67] and problem-solving strategies [68]. Specifically, participants were prompted each week to reflect on their current behaviors and areas for change, generate actionable steps to change related to the week’s lesson, identify confidence in doing so, brainstorm potential barriers, and support people. See Multimedia Appendix 1 for a screen recording of an action plan. Reminders were sent via email to participants who had not yet completed that week’s action plan 4 days after the initial email.

**Topics of weekly lessons.**

- Week 1: Overview of the Spark weight loss program (why self-monitor; losing 5% weight)
- Week 2: Green zone foods
- Week 3: Importance of physical activity
- Week 4: Reading food labels
- Week 5: Reducing sugar
- Week 6: Portion control
- Week 7: Eating out
- Week 8: Preparing meals at home; emotional eating
- Week 9: Social support; environmental cues
- Week 10: Overcoming barriers to physical activity
- Week 11: Weight loss maintenance; slippery slope; relapse prevention

#### Experimental Component

Participants were randomized to either the *detailed* arm or *simplified* arm, which varied only in their type of dietary self-monitoring.

##### Detailed Self-monitoring of Diet

Participants randomized to the *detailed* arm were instructed to self-monitor their dietary intake daily via the Fitbit mobile app. This app allows users to track their calories of all foods and beverages consumed using a built-in nutritional database, barcode scanner, or manual entry of individual recipes and to graphically view their change in caloric intake. The participants received a daily calorie goal, with a minimum of 1200 calories (kcal) per day for women and 1500 kcal per day for men, based on national guidelines [2].

##### Simplified Self-monitoring of Diet

Participants randomized to the *simplified* arm were instructed to self-monitor daily only highly caloric foods consumed that have limited nutritional value, referred to as *red zone foods*. This approach is derived from the Traffic Light Diet [69,70], which posits that limiting this category of foods will reduce intake and variety of highly caloric foods, thereby reducing the total caloric intake. Each morning, the participants in this arm were sent an automated email with a link to a brief web-based checklist (on REDCap). This checklist comprised a list of common red zone foods (ie, foods containing many calories and few nutrients). Participants were asked to select the red zone foods eaten the previous day from the list. Foods were grouped by category (eg, beverages, meat or poultry, and desserts or sweets) for easy navigation, and other red zone foods could be entered if they did not fit any item on the checklist. A goal of eating no more than 3 to 5 red zone foods per day was provided. No calorie goal was provided. Completion of this task was expected to take 2 to 5 minutes per day, compared with an estimated 34 minutes per day for tracking the calories of all foods eaten [71].

The purpose of using a simplified dietary self-monitoring strategy is to reduce the burden of self-monitoring one’s dietary intake and reduce health literacy and numeracy barriers, with the hope that this would extend engagement over longer periods while still remaining potent or impactful enough to promote healthy dietary change and subsequent weight loss.

### Measures

#### Baseline Characteristics

In the baseline survey, we collected data on participant sociodemographics, clinical characteristics (eg, smoking status, prediabetes, type 2 diabetes, or hypertension), and type of smartphone. The frequency of self-monitoring in the month before study enrollment was assessed separately for weight, steps, and diet (ie, tracking calories) using a 7-point scale ranging from *several times per day* to *never* [72].

We assessed several psychosocial variables only at the baseline time point; these variables could be examined as potential moderators of intervention effects in a fully powered efficacy trial. In particular, health literacy was assessed via the 6-item Newest Vital Sign (NVS [73]), which was administered orally by study personnel over Zoom. Scores could range from 0 to 6, with scores of 0 to 3 indicate limited health literacy and scores of 4 to 6 indicate adequate health literacy [74]. All other psychosocial measures were administered through a survey. The occurrence of 16 negative life events (eg, death of a close relative or loss of job) over the past 12 months was assessed, and a composite score was created with a possible range of 0 to 16, with higher scores indicating a greater number of negative life events experienced. Weight bias internalization, which occurs when applying negative stereotypes about one’s weight to oneself resulting in self-critical thoughts, was assessed using the modified 10-item Weight Bias Internalization Scale (WBIS [75,76]); possible scores ranged from 0 to 7, with higher scores indicating greater weight bias internalization. Sleep quality was assessed using the Pittsburgh Sleep Quality Index (PSQI [77]); a composite score was created with a possible range from 0 to 21, with higher scores reflecting worse sleep quality. A categorical measure was also created that classified scores >5 as *poor sleep* and those ≤5 as *good sleep*. Social support for eating habits was assessed by asking 10 questions each about support from family and friends [78]; for each, two 5-item subscales related to encouragement and discouragement were created, each with a possible range from 5 to 25, with higher scores indicating more encouragement or discouragement. Similarly, social support for exercise was assessed using 10 questions each about participation from family and friends [78]; for each, a composite score was created with possible scores ranging from 10 to 50, with higher scores indicating greater support.

#### Feasibility Outcomes

##### Overview

Our primary outcomes pertained to assessing the feasibility of the study procedures and engagement with the intervention. Before the start of the study, we set benchmarks that, if met, would indicate successful feasibility; these are preregistered on AsPredicted [47]. If benchmarks were not met, we brainstormed the reasons why and possible solutions for modifying the study procedures or intervention components.

##### Reach

Reach was assessed by examining the enrollment rate (ie, the proportion of participants who were enrolled and randomized out of the individuals who were eligible after completing the web-based screen) as well as by evaluating the number of participants recruited from each recruitment strategy (eg, Nextdoor, ResearchMatch, and the recruitment speed, ie, the number of participants enrolled per week). We also assessed the reasons for ineligibility among those who took the web-based screen.

##### Retention

The retention rate was operationalized as the percentage of participants who submitted a weight entry via the weight check-in survey at 1 month and 3 months and was compared with our a priori benchmark of 80% retention. We also assessed whether completers differed from noncompleters in terms of baseline sociodemographic, clinical, or psychosocial measures.

##### Survey and Dietary Recall Completion

The rate of completion of the web-based survey, as well as of the dietary recalls, was assessed and compared with our a priori benchmark of 80% completion at each time point.

##### Self-monitoring Engagement

We used Fitabase to retrieve real-time, objective, self-monitoring data on steps, body weight, and calories. We examined the median and IQR of the percentage of days of self-monitoring weight, steps, and diet over the course of the 84-day intervention. For the *simplified* arm, the self-monitoring diet was operationalized as submitting the red zone foods checklist on a given day (via a REDCap survey), whereas for the *detailed* arm, it was operationalized as a self-monitoring diet via the Fitbit app, counting only days with ≥800 calories recorded, a threshold used in previous app-based studies [19,20,25]. Each self-monitoring engagement metric was compared with our a priori benchmark of self-monitoring 75% of intervention days. Engagement in self-monitoring steps was operationalized as wearing the Fitbit activity tracker and recording at least 1 step on a given day.

In post hoc analyses, we assessed whether any participant self-monitored 100% of the days or 0% of the days. We assessed contamination by examining the number of participants who self-monitored something they were not instructed to self-monitor, which was operationalized as the number of *simplified* arm participants who self-monitored their diet via the Fitbit app (even though they were supposed to self-monitor their diet only via the red zone foods checklist).

##### Other Intervention Engagement: Action Plans

We objectively assessed action plan completion via Qualtrics and examined the median (IQR) percentage of action plans completed, with 100% indicating completion of all 11 weekly action plans. We also assessed the percentage of participants who received a reminder to complete the action plan each week and the overall reminder success rate (completion rate among those who received the reminder).

##### Other Intervention Engagement: Lessons

Using self-report in the 3-month survey, we asked participants to indicate which lessons they read and to indicate up to 3 lessons that they found most helpful. We examined the median (IQR) of lessons read.

##### Other Intervention Engagement: Feedback Emails

Using self-report in the 3-month survey, participants reported the frequency with which they read their progress reports, with 4 response options, including *weekly*, *less than 1 time per week*, *less than 1 time per month*, and *never*.

##### Other Feasibility Metrics: Timing of Baseline Procedures

To characterize the length of the recruitment and baseline period, we assessed the mean number of days elapsed between the web-based eligibility screen, remote baseline visit, and randomization.

##### Other Feasibility Metrics: Survey Time and Modality

To provide an estimate of participant burden, we assessed the median number of minutes it took to complete the 3 surveys and dietary recalls. At each time point, we asked participants to report the type of device (eg, laptop, desktop, tablet, or mobile phone) on which they completed the survey.

#### Acceptability Outcomes

We assessed the acceptability of the intervention through a series of questions in the 3-month survey, including “Would you recommend the Spark weight loss program to a friend who is trying to lose weight?” with *yes* and *no* response options. We assessed satisfaction with the Fitbit app for self-monitoring foods (shown to the *detailed* arm) or with the web-based checklist for self-monitoring red zone foods (shown to the *simplified* arm), with 6 responses ranging from *extremely dissatisfied* to *extremely satisfied* and an additional response option for *I never tracked my foods*. To understand the comprehensiveness of the Fitbit nutrition database and the list of red zone foods, we asked how likely the Fitbit app (for the *detailed* arm) or the red zone foods checklist (for the *simplified* arm) was to have the foods they typically eat, with 6 response options ranging from *extremely unlikely* to *extremely likely*. We also assessed the helpfulness of the 10 intervention components (listed in the *Results* section), with 5 response options ranging from *not at all helpful* to *extremely helpful*.

#### Exploratory Outcomes

##### Weight Change

Weight change from baseline to 1 and 3 months was assessed separately for each treatment arm. We also examined the proportion of participants who achieved clinically significant weight loss of ≥3% or ≥5% from baseline by 3 months [2,79], as well as the proportion of participants who achieved ≥2% weight loss by 1 month, which has been considered an indicator of early success in past research [80-82].

##### Caloric Intake

We examined changes in caloric intake using the Automated Self-Administered 24-hour (ASA24) Dietary Assessment Tool (version 2020), which is a free web-based tool developed by the National Cancer Institute [83]. We asked participants to complete a total of four 24-hour dietary recalls, including 2 at baseline and 2 at 3 months (for each time point, 1 on a weekday and 1 on a weekend day). Dietary recalls were available in English or Spanish (1 participant completed them in Spanish). We sent up to 4 reminders via email or SMS text message per time point to request the completion of these recalls. We excluded recalls with outliers of daily caloric intake reported as <600 kcal or >4400 kcal for women and <650 kcal or >5700 kcal for men [83]. To calculate caloric intake at each time point, we calculated the mean of the weekday recall and the weekend-day recall; if only one recall was available at a given time point, we used that value.

##### Physical Activity

We collected both a self-report and an objective measure of physical activity.

The GLTEQ self-report measure [60,61] assesses the frequency of different types of exercise (strenuous, moderate, and mild or light) for more than 15 minutes during one’s free time during the past week. Strenuous activities were described as those where one’s “heart beats rapidly” (eg, strenuous: running, jogging, or swimming), moderate activities such as “not exhausting” (eg, fast walking or tennis), and mild activities such as “minimal effort” (eg, yoga or easy walking). A leisure score index was then created using the following formula: (strenuous × 9) + (moderate × 5) + (light × 3), with higher scores indicating more frequent exercise. To assess weekly moderate to vigorous physical activity (MVPA), a composite score was created using the same procedures but excluding the light activities; from this MVPA score index, scores of ≥24 units were interpreted as *active* and scores <24 were considered *insufficiently active* [84].

We assessed step count objectively using the Fitbit Inspire 2 activity tracker. We operationalized the baseline step count as the average of the first 7 days of the intervention (week 1) and 3-month step count as the average of the last 7 days (week 12). Fitbit activity tracker devices have shown acceptable to excellent validity for step measurements [85].

##### Psychosocial Factors

Seven psychosocial measures were assessed at multiple time points via a survey.

Self-efficacy for dietary change was assessed using the 8-item Weight Efficacy Lifestyle Questionnaire Short-Form (WEL-SF [86]); possible scores ranged from 0 to 80, with higher scores indicating greater self-efficacy for making changes to one’s eating behavior in a variety of contexts, such as when tired or when in a social situation. Self-efficacy for exercise was assessed using the 12-item Self Efficacy and Exercise Habits Survey [87], possible scores ranged from 1 to 5, with higher scores reflecting greater self-efficacy for exercising in situations such as “after a long, tiring day at work.”

Three facets of motivation were assessed via the 15-item Treatment Self-Regulation Questionnaire (TSRQ) [88] with the prompt “The reason I want to achieve a healthier weight is...” The Amotivation subscale assesses the degree of lack of motivation, the Controlled Motivation subscale assesses the extent of feeling guilty or feeling external pressure to achieve this goal, and the Autonomous Motivation subscale assesses internal motivation for doing so. All subscales ranged from 1 to 7, with higher scores indicating greater levels of that construct.

Self-regulation for eating was assessed using the 18-item Three-Factor Eating Questionnaire-R18 (TFEQ-R18) [89]; 3 subscales assessed cognitive restraint (consciously restricting food intake), uncontrolled eating (feeling out of control when eating), and emotional eating (eating in response to negative emotions), with composite scores ranging from 0% to 100%; higher scores were reflective of greater levels of that construct.

Perceived stress was assessed using the 10-item Perceived Stress Scale (PSS-10) [90]; possible scores ranged from 0 to 40, with higher scores indicating greater perceived stress over the last month.

Outcome expectations were assessed at baseline and 1 month via a 17-item measure [91] of the extent to which individuals expected 16 various factors (eg, energy and eating habits) to change over the next 3 months due to participation in the Spark weight loss program. Participants also selected the 1 benefit that is most important to them. Outcome realizations were assessed at 3 months via a 17-item measure [91] that assessed the degree to which individuals felt that these 16 factors had changed owing to participation in the weight loss program. Possible scores of the composite ranged from 0 to 160, with higher scores indicating higher expectations about (or higher success of) the weight loss program.

Finally, self-efficacy for self-monitoring dietary intake was assessed at only 1 month since it could serve as a mediator of intervention effects. We administered a 14-item measure with the prompt: “a number of situations are described below that can make it hard to [track your food] (*shown to Detailed arm*) OR [track your Red Zone Foods] (*shown to Simplified arm)* on a daily basis” in contexts such as “during weekends” and “during vacations” [92]; possible scores ranged from 0% to 100%, with higher scores indicative of greater self-efficacy in self-monitoring one’s dietary intake.

### Statistical Analysis

To align with recommendations for conducting pilot studies [93-95], the purpose of our Spark Pilot Study was to assess the feasibility and acceptability of interventions and study procedures. As a result, no power analysis was conducted. The sample size of 38 participants was selected to obtain data on recruitment, randomization, and retention procedures while meeting the budget and timeline constraints. Given the higher costs than those anticipated of a study device and software, we reduced our sample size from 40 to 38 participants.

We used descriptive statistics to assess baseline characteristics, reach, feasibility outcomes, and acceptability outcomes, stratified by treatment arm, and compared them to our a priori feasibility benchmarks, when applicable. We assessed patterns of self-monitoring engagement over time by treatment arm; we report engagement data using median and IQR given their nonnormal distribution. In additional exploratory analyses, we used Spearman rank correlation coefficients to examine the relationship between self-monitoring engagement and weight change over 3 months. To examine whether any baseline variables differed by retention status (completers vs noncompleters), we used Pearson chi-square tests for categorical variables, ANOVA for continuous variables, and Fisher exact tests for small cell counts.

To capture within-arm weight change over time, we used intent-to-treat, linear mixed models via SAS PROC MIXED (SAS Institute) with an unstructured covariance matrix and restricted maximum likelihood estimates while assuming missing data at random; this approach is intended to be used in a future efficacy trial where weight change is the primary outcome. We used chi-square tests to assess the proportion of participants achieving clinically significant weight loss (≥3% and ≥5% at 3 months; ≥2% at 1 month); we used an intent-to-treat approach that included all participants. Noncompleters (ie, those who were missing a weight value at a relevant assessment time point) were assumed to have not met the clinical threshold. All other secondary outcomes were reported descriptively via change scores over time among completers. As this study was not designed to assess efficacy, we did not report between-group differences in any outcome, as recommended [93,94]. The analyses were conducted using SAS Studio (SAS Institute). Given the study’s small sample size and time constraints, we updated our preregistered protocol and no longer examined potential baseline moderators of intervention effects or conducted qualitative interviews with a subset of participants. The data analysis was conducted in 2022.

## Results

### Overview

**Figure 2:**
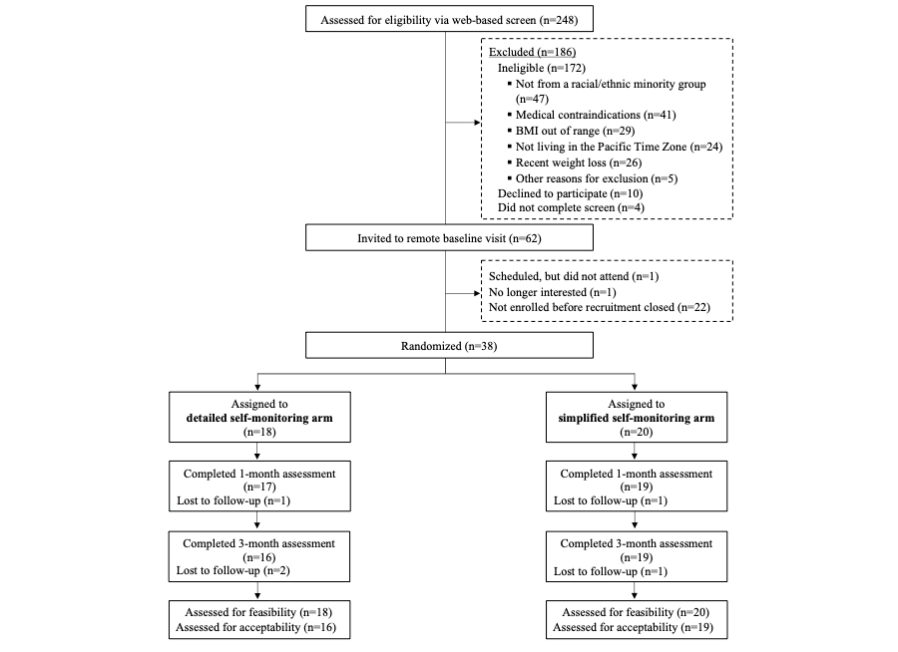
Consolidated Standards of Reporting Trials (CONSORT) flow diagram.

Figure 2, the CONSORT diagram [46], illustrates the flow of participants through the Spark Pilot Study. The web-based eligibility screen was taken by 248 individuals, of whom 25% (n=62) were eligible and invited to the remote baseline session. We enrolled 38 participants, 18 of whom were randomized to the *detailed* arm and 20 randomized to the *simplified* arm. Randomization for the study began on May 31, 2021, and data collection ended on October 8, 2021.

### Baseline Characteristics

By design, all the participants self-identified as members of US racial or ethnic minority groups. Specifically, 58% (22/38) of our sample was Hispanic of any race, 32% (12/38) were non-Hispanic Asian or Native Hawaiian or Pacific Islander, 8% (3/38) were non-Hispanic Black, and 2% (1/38) were non-Hispanic American Indian or Alaska Native (see Table 1 for all baseline characteristics).

Participants lived in 4 US states in the western region of the country and were predominantly women (32/38, 84%) with an age of 47.4 (SD 14.0; range 23-78) years and mean BMI of 31.2 (SD 4.8) kg/m^2^. In the month before the start of the study, 68% (26/38) of participants were self-monitoring steps to some degree and 87% (33/38) were self-monitoring weight, whereas only 34% (13/38) were self-monitoring dietary intake. Approximately one-quarter (11/38, 29%) of participants reported having prediabetes or type 2 diabetes and a similar (9/38, 24%) percentage had hypertension. The most important benefit participants marked as hoping to achieve from the intervention was *physical shape and appearance* (17/38, 45%), *confidence and well-being* (6/38, 16%), and *weight* (5/38, 13%). Most (31/38, 82%) participants had adequate health literacy, and almost half (18/38, 47%) reported poor sleep.

**Table 1:** Baseline demographic, clinical, and psychosocial characteristics of Spark Pilot Study participants.

				Total (N=38)		Detailed self-monitoring (n=18)		Simplified self-monitoring (n=20)
**Demographic and clinical variables**								
	Age (years), mean (SD)		47.4 (14.0)		44.6 (12.5)		49.9 (15.2)	
	Weight (kg), mean (SD)		81.2 (14.9)		83.6 (16.8)		79.0 (13.1)	
	BMI (kg/m^2^), mean (SD)		31.2 (4.8)		32.1 (4.8)		30.5 (4.8)	
	**BMI category, n (%)**							
		Overweight (25-29.9 kg/m^2^)	18 (47)		8 (44)		10 (50)	
		Obesity (30-45.0 kg/m^2^)	20 (53)		10 (56)		10 (50)	
	**Gender, n (%)**							
		Man	6 (16)		2 (11)		4 (20)	
		Woman	32 (84)		16 (89)		16 (80)	
	**Marital status, n (%)**							
		Married or living with partner	26 (68)		9 (50)		17 (85)	
		Not married or living with partner	12 (32)		9 (50)		3 (15)	
	**Race and ethnicity, n (%)**							
		Hispanic (any race)	22 (58)		11 (61)		11 (55)	
		Non-Hispanic Asian or Native Hawaiian or Pacific Islander	12 (32)		7 (39)		5 (25)	
		Non-Hispanic Black	3 (8)		0 (0)		3 (15)	
		Non-Hispanic American Indian or Alaska Native	1 (3)		0 (0)		1 (5)	
	**Education, n (%)**							
		Less than college graduate	16 (42)		6 (33)		10 (50)	
		College graduate (4 years) or more	22 (58)		12 (67)		10 (50)	
	**Employment status, n (%)**							
		Employed	28 (74)		15 (83)		13 (65)	
		Not employed	10 (26)		3 (17)		7 (35)	
	**Annual household income (US $), n (%)**							
		0-49,999	5 (13)		3 (17)		2 (10)	
		50,000-99,999	13 (34)		6 (33)		7 (35)	
		100,000 or greater	18 (47)		7 (39)		11 (55)	
		Unknown	2 (5)		2 (11)		0 (0)	
	**Self-monitoring of diet frequency before enrollment, n (%)**							
		Daily	0 (0)		0 (0)		0 (0)	
		1 to 6 times per week	4 (11)		2 (11)		2 (10)	
		<1 time per week	9 (24)		5 (28)		4 (20)	
		Never	25 (64)		11 (61)		14 (70)	
	**Self-monitoring of weight frequency before enrollment, n (%)**							
		Daily	5 (13)		1 (6)		4 (20)	
		1 to 6 times per week	14 (37)		5 (28)		9 (45)	
		<1 time per week	14 (37)		8 (44)		6 (30)	
		Never	5 (13)		4 (22)		1 (5)	
	**Self-monitoring of steps frequency before enrollment, n (%)**							
		Daily	13 (34)		2 (11)		11 (55)	
		1 to 6 times per week	7 (18)		6 (33)		1 (5)	
		<1 time per week	6 (16)		2 (11)		4 (20)	
		Never	12 (32)		8 (44)		4 (20)	
	**Smoking status, n (%)**							
		Never smoker	33 (87)		15 (83)		18 (90)	
		Former smoker	3 (8)		1 (6)		2 (10)	
		Current smoker	2 (5)		2 (11)		0 (0)	
	Prediabetes or type 2 diabetes, n (%)		11 (29)		7 (39)		4 (20)	
	Hypertension, n (%)		9 (24)		5 (28)		4 (13)	
	**Type of smartphone, n (%)**							
		iPhone	25 (66)		13 (72.2)		12 (60)	
		Android	13 (34)		5 (27.8)		8 (40)	
**Psychosocial variables**								
	Limited health literacy (NVS^a^), n (%)		7 (18)		2 (11)		5 (25)	
	Negative life events in past 4 months, mean (SD)		2.5 (2.6)		2.9 (3.1)		2.1 (2.1)	
	Weight bias internalization (WBIS^b^), mean (SD)		3.5 (1.6)		4.3 (1.4)		2.7 (1.3)	
	**Sleep quality (PSQI^c^), mean (SD)**		6.4 (3.7)		6.9 (3.8)		6.1 (3.7)	
		Poor sleep, n (%)	18 (47)		11 (61)		7 (35)	
	**Social support for eating habits (SSEH^d^), mean (SD)**							
		Encouragement—family	11.2 (4.9)		10.9 (4.5)		11.5 (5.3)	
		Encouragement—friends	8.3 (4.6)		8.5 (4.8)		8.1 (4.5)	
		Discouragement—family	11.9 (3.8)		12.4 (4.2)		11.5 (3.5)	
		Discouragement—friends	8.3 (3.4)		8.2 (3.8)		8.5 (3.2)	
	**Social support for exercise (SSES^e^), mean (SD)**							
		Family participation	25.9 (12.3)		21.5 (10.7)		29.8 (12.6)	
		Friend participation	17.8 (8.4)		17.4 (7.7)		18.2 (9.2)	

^a^NVS: Newest Vital Sign.

^b^WBIS: Weight Bias Internalization Scale.

^c^PSQI: Pittsburgh Sleep Quality Index.

^d^SSEH: Social Support for Eating Habits.

^e^SSES: Social Support for Exercise Survey.

### Feasibility Outcomes

#### Overview

Table 2 provides an overview of the feasibility findings compared with our a priori benchmarks of success. Benchmarks were met for all 12 metrics in the *simplified* arm versus 9 of the 12 metrics in the *detailed* arm.

**Table 2:** Feasibility outcomes compared with a priori benchmarks.

Feasibility metric		Detailed self-monitoring (n=18)	Simplified self-monitoring (n=20)	Benchmark (%)
**Intervention Engagement over 3 months, median (IQR)**				
	Days self-monitoring weight (%)	67 (2-82)	80 (38-100)	75
	Days self-monitoring steps (%)	99 (58-100)	100 (99-100)	75
	Days self-monitoring diet via red zone foods survey (%)	N/A^a^	97 (86-100)	75
	Days self-monitoring diet via Fitbit app (%)	49 (5-67)	N/A	75
	Action plans completed (%)	95 (55-100)	100 (91-100)	80
	Lessons reviewed^b^ (%)	100 (45-100)	100 (95-100)	80
**Retention, n (%)**				
	1 month	17 (94)	19 (95)	80
	3 months	16 (89)	19 (95)	80
**Survey completion, n (%)**				
	Baseline	18 (100)	20 (100)	80
	1 month	17 (94)	19 (95)	80
	3 months	16 (89)	19 (95)	80
**Dietary recall completion, n (%)^c^**				
	Baseline	17 (94)	19 (95)	80
	3 months	12 (67)	17 (85)	80
**Weight change from baseline (kg), mean (SD; 95% CI)**				
	1 month	−1.85 (1.96; −2.79 to −0.91)	−1.59 (1.94; −2.48 to −0.71)	—^d^
	3 months	−3.41 (2.52; −4.62 to −2.20)	−3.29 (2.44; −4.41 to −2.18)	—
**Weight change from baseline (%), mean (SD; 95% CI)**				
	1 month	−2.13 (2.75; −3.45 to −0.81)	−2.07 (2.71; −3.30 to −0.83)	—
	3 months	−4.12 (2.80; −5.47 to −2.78)	−4.20 (2.71; −5.43 to −2.96)	—
**Clinically significant weight loss from baseline, n (%)^e^**				
	≥2% weight loss at 1 month	9 (50)	9 (45)	—
	≥3% weight loss at 3 months	9 (50)	14 (70)	—
	≥5% weight loss at 3 months	8 (44)	7 (35)	—

^a^N/A: not applicable. N/A because the specific arm was not assigned to self-monitor this item.

^b^Analyses assume that individuals who did not complete the 3-month survey did not review any progress reports or lessons.

^c^Participants were asked to complete 2 ASA24 dietary recalls (1 week day and 1 weekend day) at each time point. If 0 or 1 completed, it is marked as not completed for that time point.

^d^Indicates no a priori benchmark was set.

^e^Analyses assumed that individuals who did not submit a weight entry at a given follow-up time point did not achieve clinically meaningful weight loss.

#### Reach

We achieved an enrollment rate of 61% (38 randomized out of 62 eligible patients after screening). The main sources of recruitment were ResearchMatch (20/38, 53%), an institute-specific diabetes registry (14/38, 37%), and Nextdoor (4/38, 11%).

We achieved an average recruitment speed of 10 participants per week, with all participants being enrolled within 1 month. The most common reasons for ineligibility were not identifying as a racial or ethnic minority group member (47/172, 27.3%), having a medical contraindication (41/172, 23.8%), having a BMI <25 kg/m^2^ or >45 kg/m^2^ (29/172, 17%), experiencing recent weight loss of 5% (26/172, 15.1%), and not living in the US Pacific time zone (24/172, 13.9%).

#### Retention

Retention rates surpassed our a priori benchmark of 80% at both 1 month (95% retention) and 3 months (92% retention). Completers (n=35) at 3 months differed from noncompleters (n=3) on several baseline factors, including reporting fewer negative life events (P=.03) and lower perceived stress (P=.01), amotivation (P=.02), uncontrolled eating (P=.003), and discouragement (and encouragement) from friends in making dietary change (P=.004 and P=.03), as well as their history of self-monitoring dietary intake (P=.02), with 71% (25/35) of completers never tracking diet in the past month compared with 0% of noncompleters (data not shown).

#### Survey and Dietary Recall Completion

The survey completion rates met our a priori benchmark of 80% at all 3 time points for both the *detailed* and *simplified* arms. Dietary recall (ie, ASA24) completion rates met the a priori benchmark of 80% at the baseline assessment for both *detailed* and *simplified* arms, but at 3 months, only the *simplified* arm met this benchmark (85% completion vs 67% for *detailed*).

#### Self-monitoring Engagement

**Figure 3:**
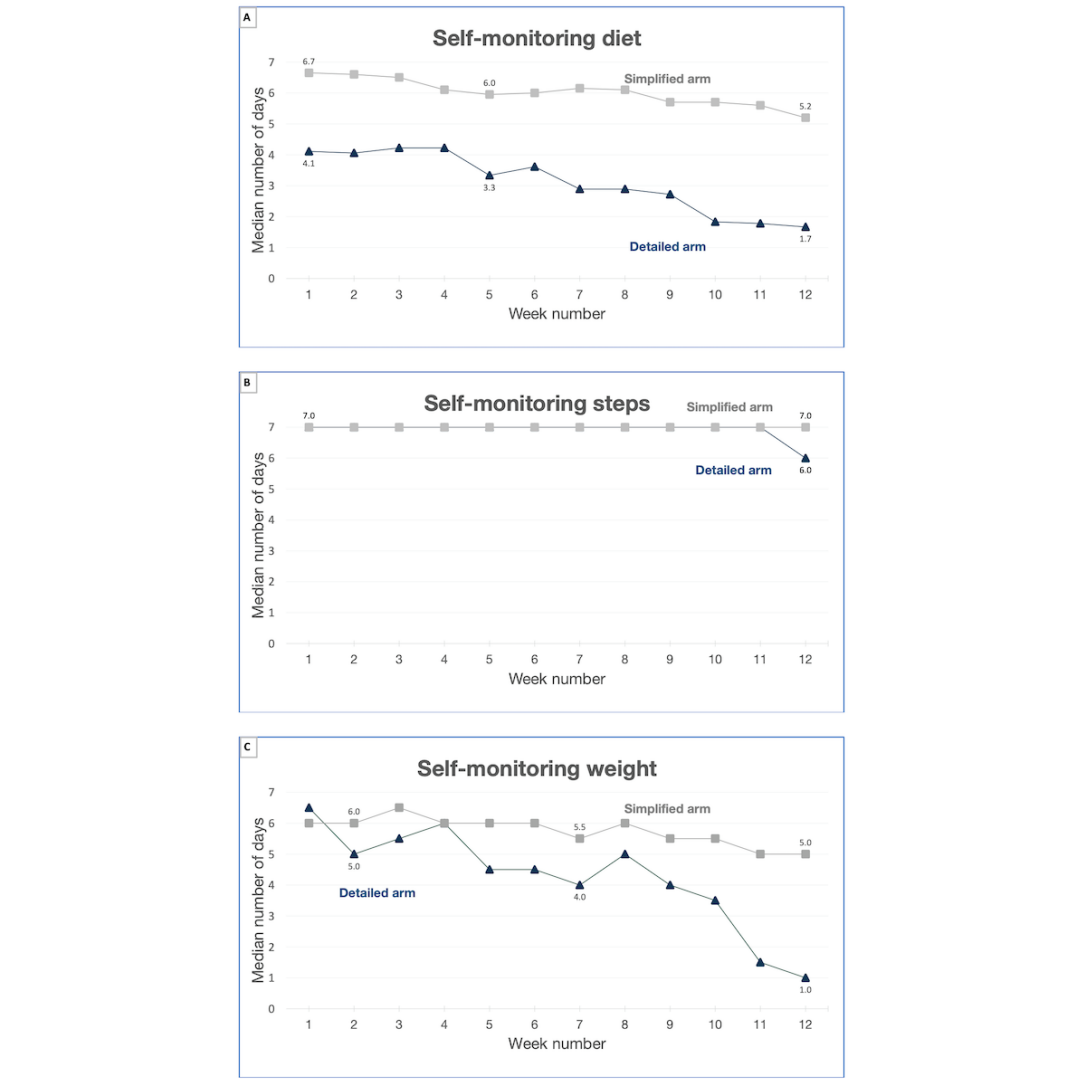
Self-monitoring engagement over the 12-week intervention by arm.

The *detailed* arm did not meet benchmarks for 2 of 3 self-monitoring metrics, with a median percent day of self-monitoring of 49% for diet (ie, all foods eaten via the Fitbit app), 67% for weight, and 99% for steps. The *simplified* arm met benchmarks for all 3 self-monitoring metrics, with a median percent day of self-monitoring of 97% for diet (ie, red zone foods via checklist), 80% for weight, and 100% for steps (see Figure 3 for engagement over time).

##### High and Low Engagement

Among all 38 participants, several self-monitored every day in the intervention, including 8 (21%) participants who did so for diet tracking, 23 (61%) for step tracking, and 7 (18%) for weight tracking. Of the 8 participants who self-monitored their diet for all days, 7 (87%) were in the *simplified* arm. In addition, some participants did not self-monitor. Specifically, in the *detailed* arm, of the 18 participants, 4 (22%) participants never tracked diet, while 3 (17%) never tracked steps, and 4 (22%) never tracked weight. Three participants did not track any domains. In the *simplified* arm, all (n=20) participants tracked diet and steps to some degree, although 1 (5%) never tracked weight.

##### Contamination

We found that 2 *simplified* arm participants self-monitored their diet via the Fitbit app (when they were instructed to track only red zone foods), one of whom did so for 9 days and then stopped, and another who did so for 99% of days. Both participants self-monitored the red zone foods for 100% of days.

#### Other Intervention Engagement

##### Action Plans

Action plan completion rates met our a priori benchmark of 80%, with a median of 95% completed in *detailed* and 100% completed in *simplified*. Among all participants, 58% (22/38) completed all 11 action plans, while only 1 participant completed none. Reminders were sent each week to participants who had not yet completed their action plans, with 9% (3/34) of participants receiving reminders for action plan 1 to 45% (17/38) receiving reminders for action plan 10. Reminders appeared to be helpful in prompting the completion of action plans, with an overall reminder success rate of 39%.

##### Lessons

The rates of lessons read met our a priori benchmark of 80%, with a median of 100% in both arms. Many participants (26/38, 68%) reported reading all 11 lessons, while no participants reported reading none (though 3 participants did not answer this question because they did not complete the 3-month survey). The lessons with the most votes for *most helpful* were on the topics of *weight loss maintenance* (34% of participants selected this in their top 3), *reading food labels* (26%), and *portion control* (26%).

##### Feedback Emails

We did not gather objective data on which feedback emails were read, but we did assess the self-reported frequency of doing so over the 3-month intervention. Many participants (14/18, 78% of the *detailed* arm; 18/20, 90% of the *simplified* arm) reported reading their progress reports weekly.

#### Other Feasibility Metrics

##### Timing of Baseline Procedures

The mean number of days elapsed between the eligibility screen and the remote baseline visit was 7 (SD 5) days. A mean of 16 (SD 4) days elapsed from the baseline visit to randomization (as participants waited for their devices to arrive in the mail).

##### Survey Time and Modality

The median number of minutes to complete the ASA24 dietary recall was 20 minutes: 29 minutes for the baseline survey, 16 minutes for the 1-month survey, and 32 minutes for the 3-month survey. Across all time points, the majority (77/109, 70.6%) of participants reported taking the survey on a laptop or desktop computer, while 24.7% (27/109) reported using a smartphone and 4.5% (5/109) reported using a tablet device. Many (21/36, 58%) participants completed the surveys on different modalities at different time points.

### Acceptability Outcomes

**Figure 4:**
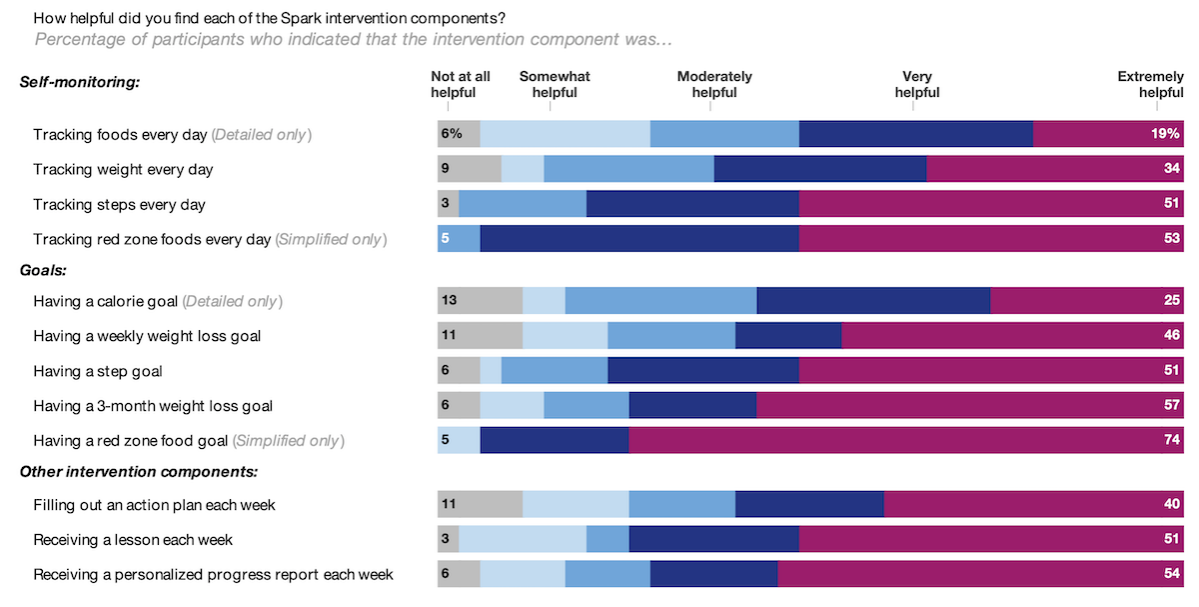
Helpfulness ratings for each intervention component. This chart depicts the participants’ satisfaction with the 10 intervention components. The proportion of participants who selected “not at all helpful” or “extremely helpful” is shown.

Most participants (34/38, 89% overall; 16/18, 88% in the *detailed* arm and 18/20, 90% in the *simplified* arm) indicated that they would recommend the Spark Pilot weight loss program to a friend who is trying to lose weight. Among survey completers (n=35), roughly half (9/16, 56%) of the participants in the *detailed* arm indicated being *somewhat* to *extremely* satisfied with the Fitbit app for tracking foods, compared with almost all (18/19, 95%) participants in the *simplified* arm who used the web-based checklist for tracking red zone foods. When asked about how likely the assigned self-monitoring platform was to have the foods participants typically eat, 38% (6/16) of participants in the *detailed* arm indicated *moderately* or *somewhat* unlikely when reflecting on the Fitbit app, compared with 11% (1/19) of participants in the *simplified* arm who reflected on the red zone foods checklist. Each of the intervention components was rated by most participants as *very* or *extremely* helpful, with “tracking red zone foods every day” rated as such by 95% of the participants in the *simplified* arm, whereas “tracking foods every day” was rated as such by 56% of the participants in the *detailed* arm (see Figure 4 for all ratings).

### Exploratory Outcomes

#### Weight Change

**Figure 5:**
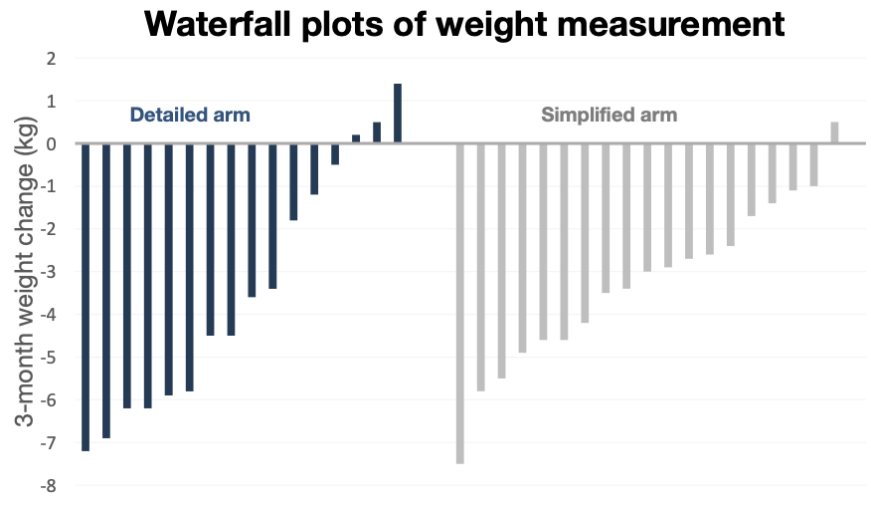
Waterfall plots showing individual participant weight change in kilograms from baseline to 3 months in the detailed arm (left) and in the simplified arm (right). Each bar represents an individual participant. Three participants did not complete the 3-month assessment and are not represented in the figure.

At 1 month, mean weight change from baseline was −1.85 (95% CI −2.79 to −0.91) kg in the *detailed* arm and −1.59 (95% CI −2.48 to −0.71) kg in the *simplified* arm. At 3 months, weight change from baseline was −3.41 (95% CI −4.62 to −2.20) kg in the *detailed* arm and −3.29 (95% CI −4.41 to −2.18) kg in the *simplified* arm. Over one-third of the participants achieved ≥5% weight loss at 3 months (8/18, 44% in the *detailed* arm; 7/20, 35% in the *simplified* arm). The percent weight change outcomes are presented in Table 2. The individual participant weight change is presented via waterfall plots in Figure 5. See Multimedia Appendix 2 for the rest of the exploratory outcomes.

#### Relation Between Self-monitoring and Weight Change

In the *detailed* arm, there were no significant associations between 3-month percent weight change and engagement in any of the self-monitoring metrics (self-monitoring steps: *r_s_*=0.08, P=.78; self-monitoring weight: *r_s_*=−0.11, P=.69; self-monitoring diet: *r_s_*=0.18, P=.52). In the *simplified* arm, the 3-month percent weight change was associated with self-monitoring weight (*r_s_*=−0.46, P=.048), but not with the other self-monitoring metrics (self-monitoring steps: *r_s_*=−0.42, P=.07; self-monitoring diet: *r_s_*=0.06, P=.80).

#### Caloric Intake

Descriptively, both the *detailed* and *simplified* arms saw reductions in caloric intake over time (−403 kcal/day and −364 kcal/day, respectively).

#### Physical Activity

In week 1, the mean step count was high (8257 steps), with a range of 2651 to 15,264 steps. Descriptively, physical activity increased in the *simplified* arm over 3 months, with a mean 15.3 (SD 12.7) unit increase in the GLTEQ leisure score index, a 24% increase in the number of participants achieving the *active* category of MVPA, and a mean increase of 250 (SD 3162) steps. These improvements were not observed in the *detailed* arm (D GLTEQ leisure score index: mean 0.9, SD 22.9; D in percentage of *active* participants: −29.5%; D steps: mean −1306, SD 2444 steps).

In a post hoc exploratory analysis of completers, those assigned to the initial 5000 step goal (n=10) had a mean of 6316 steps per day by the end of the intervention, compared with 9384 steps per day among those with the initial 7000 step goal (n=7) and 9203 steps per day among those with the initial 10,000 step goal (n=21).

#### Psychosocial Factors

Descriptively, improvements from baseline to 1 month and 3 months were found for both arms in self-efficacy for dietary change, self-efficacy for exercise, and all self-regulation subscales (Multimedia Appendix 2). Motivation scores on all 3 subscales showed little change over time. Perceived stress levels remained high throughout the intervention, whereas outcome realization scores at 3 months were lower than outcome expectations assessed at baseline and 1 month.

## Discussion

### Principal Findings and Lessons Learned

In this pilot study, we established the feasibility and acceptability of a 3-month stand-alone digital weight loss intervention in a racially or ethnically diverse sample of adults that involved either detailed or simplified dietary self-monitoring. We observed high retention, moderate to high engagement in self-monitoring, high satisfaction with the weight loss program, and clinically meaningful short-term weight loss. Importantly, we were able to reach our target population of racial and ethnic minority adults, who are underrepresented in clinical trials of behavioral weight management interventions despite having disproportionate obesity rates. We also found that using a fully remote trial design in which recruitment, intervention, and assessment procedures were all performed remotely enabled greater geographic diversity and more rapid recruitment than is typically possible in research studies involving in-person procedures. To further achieve these recruitment and retention targets, we offered an array of times, including evenings and weekend days, to meet with participants via Zoom for the baseline visit, which was the only face-to-face interaction between the study personnel and participants. All other assessments and intervention procedures were performed via email or text message communication.

The feasibility was assessed based on whether a priori benchmarks were met. Specifically, the *simplified* arm successfully met all 12 a priori feasibility benchmarks, whereas the *detailed* arm met 9 of 12 benchmarks. Domains that were not met for the latter arm include engagement rates for self-monitoring weight and diet (67% and 49% of days, respectively, vs the 75% of days benchmark) and the completion rate of the 3-month dietary recall (67% vs 80% benchmark). To guide further modifications of our intervention and provide suggestions for other studies, we have compiled challenges and proposed solutions in Table 3.

Although we observed higher engagement in dietary self-monitoring in the *simplified* arm than in the *detailed* arm (97% vs 49% of days, respectively), as well as higher satisfaction and helpfulness ratings, the magnitudes of weight loss were similar in both arms (3.3 kg in the *simplified* arm and 3.4 kg in the *detailed* arm). A larger, longer-term intervention study would provide further information on whether such differences would at some point reflect differential weight loss between the 2 arms. When designing digital strategies to promote engagement, both the perceived ease of use and usefulness of such approaches are needed, according to the Technology Acceptance Model [96] and supported by qualitative feedback [97]. It is likely that the simplified dietary self-monitoring approach resulted in high engagement because of meeting both of these needs. Concurrently, the detailed approach may have been harder to use and more time-intensive (eg, 6/16, 38% of participants in the *detailed* arm reported that the app was unlikely to have foods they typically eat, which would have required the manual creation of new foods), which likely resulted in lower engagement. However, whether such distinctions between the 2 approaches result in measurable effects across longer weight loss intervention periods remains unclear. As noted earlier, these findings are meant to be hypothesis generating, as we were not able to detect significant weight loss effects between the arms.

**Table 3:** Challenges encountered in the Spark Pilot Study and potential solutions.

Challenge			Purpose of addressing it		Potential solutions
**Did not meet a priori** *benchmark*					
	Detailed arm: engagement in self-monitoring dietary intake via Fitbit app	Suboptimal weight loss can occur with declining engagement rates over time		Use an app with a built-in reminder to track diet or set up automated reminders via a software program	
	Detailed arm: engagement in self-monitoring weight via e-scale possibly due to switching from manual entry vs automated collection of weight values	Suboptimal weight loss can occur with declining engagement rates over time		Use the smart scale features of a scale to enhance ease of use and avoid manual entry of weight data into an app; request institutional review board approval for multiple scales before the start of recruitment if purchasing devices via a consumer website	
	Detailed arm: completion of dietary recalls	To gain a more accurate understanding of dietary change		Set clear expectations of time and purpose of the recall; provide greater incentives for completion; drop or switch to a simpler recall	
**Other issues identified**					
	Treatment contamination: 2 simplified arm participants self-monitored all foods eaten via Fitbit app (while also self-monitoring red zone foods)	Minimizes ability to detect differences in feasibility, acceptability, and (eventually) efficacy between conditions when participants engage in intervention content intended for a different condition		Clarify upfront participants’ understanding of what to do and what not to do as part of their intervention. State the importance of adhering to the intervention content of one’s condition and not engaging in activities intended for other conditions. In addition, conduct interviews with participants to understand reasons for engaging in these activities.	
	Three participants never self-monitored any domain	It is helpful to uncover the reason for no engagement (eg, owing to technical issues, unclear expectations set, life circumstances, or other)		Provide additional support from study personnel for setting up devices within 1 to 2 days of randomization if not yet synchronizing; set up prompts through a variety of channels to check in on whether any technical issues have been encountered; set clear expectations before enrollment of time commitment	
	Some confusion about when to start the weight loss program	To avoid dissatisfaction and dropout		During the baseline visit, provide an estimate of the average time before starting the weight loss program to receive the devices (in our study, a mean of 16 days elapsed owing to time for personnel to order devices and delivery time); explain that devices might arrive on different days, and they should wait until receiving an email to begin the weight loss program	

### Comparison With Prior Work

In total, 4 of the 5 prior RCTs comparing detailed versus simplified self-monitoring approaches [25,26,28,29] found similar weight loss between arms at the end of the intervention; the other trial observed greater weight loss in the detailed arm [27]. In all 5 trials, engagement rates in dietary self-monitoring were similar between arms, which is contrary to our finding that engagement rates appeared meaningfully higher in the *simplified* arm than in the *detailed* arm. These differences may be explained by the specific type of simplified dietary self-monitoring approach that we tested (ie, a web-based checklist of red zone foods). It is likely that participants found this strategy particularly easy to use, which is supported by our finding of high ratings of satisfaction and helpfulness. To aid in ease of use, the *simplified* arm had a daily reminder to track their red zone foods by way of receiving an automated email from REDCap each morning to complete their checklist. The use of reminders has been noted as a helpful strategy in promoting self-monitoring engagement [98]. Among prior weight loss studies, the only other interventions with high engagement (>80%) in simplified dietary self-monitoring strategies, to our knowledge, included those that assessed rates on a weekly rather than daily time frame [33,36], although many studies did not report engagement rates. Among all of these trials, the results should be interpreted with caution because of their small sample sizes or unreported engagement rates.

Engagement in dietary self-monitoring in the *detailed* arm (49% of days) was comparable with engagement rates in other weight loss trials; a 2021 systematic review found that 58% (11/19) of interventions with dietary self-monitoring achieved average engagement rates of ≥50% of days [13]. It is possible that incorporating reminders to track diet at the end of the day if not yet done could enhance engagement, as suggested by our previous trial that found a median of 77% (IQR 27%-96%) days of self-monitoring in a 3-month intervention that used a commercial app (MyFitnessPal) that had built-in reminders to track diet [19]. The addition of these reminders should be tested empirically.

Compared with other stand-alone digital weight loss interventions, the magnitude of weight loss found in our study was comparable with many studies [10,25,27,29,36,99], higher than some [26,41,42,100], and lower than a recent fully automated, large-scale pragmatic trial comprising mostly non-Hispanic White participants [101]. This is an important area of research, as many barriers exist to attending in-person treatment, such as living far from a medical clinic or treatment program [102], having childcare or caregiver responsibilities, or having fluctuating working schedules. In addition, not all individuals who want to lose weight are ready or able to commit to treatment during weekly counseling sessions. Thus, stand-alone interventions have the potential to meet the weight loss needs of many individuals by producing modest weight loss [10], while simultaneously lowering the intensity of participation and offering greater scalability and reach than interventions with counselor support, given widespread accessibility and lower personnel costs. Digital interventions have also produced reductions in attrition in clinical trials [103], likely because they afford participants greater flexibility in engaging in treatment and impose fewer transportation and time constraints. With the widespread uptake of smartphones among US adults, including among racial and ethnic minorities (eg, 79% among Hispanic or Latinx adults and 80% among Black adults [104]), stand-alone digital health interventions have the potential to reach broad populations who may otherwise not be able to access behavioral obesity treatment.

To our knowledge, few past studies on behavioral weight management interventions have been conducted entirely remotely [28,105,106]. Two recent studies switched from an in-person to a fully remote format due to the COVID-19 pandemic and reported how outcomes differed by format: the weight loss maintenance study by Leahey et al [45] observed higher treatment attendance when done remotely versus in-person and excellent retention at study assessments for up to 18 months in both formats. They found that Hispanic participants, in particular, preferred remote sessions. In a 16-week weight loss intervention conducted by Ross et al [107], similar high levels of weight loss and self-monitoring engagement were achieved between a completely remote cohort and a cohort that started in person and transitioned to a remote setting at 11 weeks.

### Strengths and Limitations

A strength of the Spark Pilot Study is the focus on racial and ethnic minority adults who are underrepresented in clinical trials of behavioral weight management interventions despite having disproportionate obesity rates. Other strengths include high retention, collection of objective self-monitoring data via digital tools, and use of an RCT design to assess the acceptability and feasibility of the intervention and of study procedures, as well as the successful implementation of a fully remote trial format (spanning from recruitment, onboarding during the baseline visit, mailing devices to participants’ homes, randomization, assessments, and intervention delivery) that will be replicated in a future efficacy trial. As the COVID-19 pandemic has prompted other researchers to swiftly transition their in-person research studies to remote platforms, we hope that our study will serve as one example for how to leverage remote technologies and project management tools.

This study has several limitations. First, we were unable to use and test the smart scale features of the Withings scale we provided to participants because of a global chip shortage that made the commercial scale unavailable, followed by institutional review board delays in approving a different commercial scale. Second, given the limited number of personnel, the principal investigator and study staff were not blinded to treatment allocation. However, because the intervention was stand-alone, the study team did not interact face-to-face with the participants beyond the baseline visit, and automated surveys and templated email messages were used to collect weight and other outcomes. Third, we did not capture metrics for ease of use or perceived burden or time intensity of the 2 dietary self-monitoring approaches, although questions on satisfaction and helpfulness, as well as the objective engagement rates help inform the acceptability of these approaches. Fourth, we recruited only 3 non-Hispanic Black participants, all of whom were randomized to the simplified arm. Further efforts to reach this demographic, who face the highest obesity prevalence, should be considered. Relatedly, only 16% of the participants were men, with twice as many randomized to the simplified arm than the detailed arm. Recruiting a larger sample size and stratifying by gender should help to minimize the potential for gender to be a confounder in a future efficacy trial. Fifth, because of the pilot study’s focus on feasibility and acceptability and small sample size, we did not assess outcomes by race and ethnicity status, which would be an important step in a larger efficacy trial to determine if results vary by race or ethnicity. Finally, contamination occurred among 2 of the simplified arm participants in that they also self-monitored caloric intake when instructed not to do so; minimizing contamination will be particularly important in a future efficacy trial because it could impact the magnitude of weight change and lead to an inaccurate interpretation of findings.

### Future Research

With the feasibility and efficacy established and minimal refinements needed, a fully powered efficacy trial can now be conducted to test between-group differences in weight loss between the *detailed* and *simplified* self-monitoring arms. This future trial would replicate most of the study procedures used in this pilot study; the main changes would include extending the intervention length, recruiting nationwide in the United States, expanding the sample size to have sufficient power to examine efficacy outcomes, and investigating moderators of treatment response, such as whether certain racial and ethnic groups respond better to a simplified versus detailed self-monitoring approach. None of the prior 5 RCTs that compared detailed to simplified self-monitoring enrolled more than 100 participants (mean 60), and none extended beyond 6 months; thus, trials with larger sample sizes and longer trial durations are needed to more clearly assess efficacy, moderators of treatment response, and maintenance of weight loss. One trial, AGILE, is currently being conducted among 608 young adults testing a detailed versus simplified dietary self-monitoring approach as part of a factorial design in a mobile health intervention (ClinicalTrials.gov: NCT04922216). The findings from this study will advance our understanding of which approach is more suitable for a young adult population.

It is also important to replicate this research question in the context of a more intensive behavioral weight loss treatment that involves frequent counseling [2]. Although self-monitoring engagement rates were found in a systematic review to be similar in stand-alone interventions versus those with counseling [13], it is possible that empirically testing this question in an intensive treatment context would result in a different conclusion. It would also be worthwhile to continue to develop and test new simplified dietary self-monitoring approaches, such as using artificial intelligence to estimate the caloric intake of foods [108], which may increase accuracy while decreasing burden; comparing different simplified approaches to one another in a clinical trial (eg, self-monitor red zone foods vs dietary lapses vs photos of food) is also needed to determine the strategy with the greatest engagement, highest satisfaction, and largest magnitude of weight loss. Furthermore, using a simplified dietary self-monitoring approach for the duration of an intervention versus using it only if low engagement in detailed dietary self-monitoring occurs is another area to be empirically tested [23,24]. Finally, whether both passive and active forms of self-monitoring impact weight loss similarly should be investigated, given that passive forms of monitoring (eg, wearing an activity tracker) may decrease burden by leveraging automatic data collection from a device, whereas active forms of self-monitoring require the user to volitionally record information somewhere.

Taken together, continuing to enhance the potency of digital weight loss interventions is advantageous to address the obesity epidemic on a large scale. Evaluating different intensities, frequencies, and formats of dietary self-monitoring will help refine these weight loss programs and ultimately maximize clinically meaningful weight loss.

### Conclusions

Given that the positive impacts of dietary self-monitoring on weight loss have been repeatedly demonstrated [13,14,109,110], what needs to follow is how to prolong self-monitoring engagement to enhance and sustain weight loss. Simplified dietary self-monitoring strategies are explicitly designed to be easier to use than detailed approaches, but a question remains as to whether they can be potent enough to produce clinically meaningful weight loss. We found that a simplified self-monitoring strategy consisting of tracking only foods that are high in calories and low in nutritional value via a daily web-based checklist resulted in high engagement, high ratings of acceptability, and clinically meaningful weight loss—indicators that both perceived ease of use and usefulness were achieved. These findings were obtained in the context of a fully remote intervention among racial and ethnic minority adults. This pilot study laid the foundation for conducting a long-term, fully powered trial to compare the efficacy of a simplified versus detailed dietary self-monitoring approach in this context. If deemed effective in a subsequent efficacy trial, this lower-intensity stand-alone intervention has the potential to serve as a first-line treatment strategy for this population. In addition, fully remote study procedures could serve as a model for researchers seeking to broaden their reach and access to similar behavioral interventions.

## Appendix

Multimedia Appendix 1 Screen recording of an example action plan.

Multimedia Appendix 2 Exploratory outcomes over time, by treatment arm.

Multimedia Appendix 3 CONSORT-eHEALTH checklist (V 1.6.1).

## Abbreviations

ASA24: Automated Self-Administered 24-Hour
CONSORT: Consolidated Standards of Reporting Trials
GLTEQ: Godin Leisure-Time Exercise Questionnaire
MVPA: moderate to vigorous physical activity
NVS: Newest Vital Sign
PAR-Q+: Physical Activity Readiness Questionnaire for Everyone
PSQI: Pittsburgh Sleep Quality Index
PSS-10: 10-item Perceived Stress Scale
RCT: randomized controlled trial
REDCap: Research Electronic Data Capture
TFEQ-R18: 18-item Three-Factor Eating Questionnaire-R18
TSRQ: Treatment Self-Regulation Questionnaire
WBIS: Weight Bias Internalization Scale
WEL-SF: Weight Efficacy Lifestyle Questionnaire Short-Form

## Data Availability

The data set used in this study is available from the corresponding author (MLP) upon reasonable request.
