# Supplementary material for: Detailed Versus Simplified Dietary Self-monitoring in a Digital Weight Loss Intervention Among Racial and Ethnic Minority Adults: Fully Remote, Randomized Pilot Study
Source: JMIR Form Res. 2022 Dec 13;6(12):e42191. doi: 10.2196/42191 (PMC9795401; doi:10.2196/42191)
Supplement: Multimedia Appendix 2 [file formative_v6i12e42191_app2.pdf]

Multimedia Appendix 2

Patel ML, Cleare AE, Smith CM, Rosas LG, & King AC. Detailed Versus Simplified Dietary Self-monitoring in a Digital Weight Loss Intervention Among Racial and Ethnic Minority Adults: Fully Remote, Randomized Pilot Study. *JMIR Form Res* 2022;6(12):e4219.1
URL: https://formative.jmir.org/2022/12/e42191/
doi: 10.2196/42191

Supplemental Table. Exploratory outcomes over time, by treatment arm.[a]

	Detailed Self-Monitoring (n=18)			Simplified Self-Monitoring (n=20)		
	Baseline	1 Month	3 Months	Baseline	1 Month	3 Months
**Behavior Change Outcomes**						
Caloric intake, mean (SD), kcal[b]	2017 (812)	--[c]	1336 (527)	1874 (498)	--	1458 (494)
Physical activity (GLTEQ)						
leisure score index, mean (SD)	26.8 (15.7)	28.9 (21.3)	27.1 (22.8)	33.1 (31.3)	43.1 (25.5)	51.1 (27.4)
MVPA score index, mean (SD)	18.3 (14.6)	15.9 (18.0)	16.8 (19.7)	22.7 (21.5)	30.1 (22.2)	39.8 (24.6)
Active, No. (%)	8 (44.4)	5 (29.4)	5 (31.3)	11 (55.0)	14 (73.7)	13 (68.4)
Insufficiently active, No. (%)	10 (55.6)	12 (70.6)	11 (68.8)	9 (45.0)	5 (26.3)	6 (31.6)
Step count (Fitbit), mean (SD)[d]	7773 (3625)	8222 (4227)	6882 (3975)	8619 (3276)	8534 (3750)	9219 (4204)
**Psychosocial Factors, mean (SD)**						
Self-efficacy for dietary change (WEL-SF)	39.4 (15.5)	52.4 (16.3)	52.5 (17.1)	47.5 (11.9)	53.2 (16.2)	59.9 (12.9)
Self-efficacy for exercise	3.3 (0.8)	3.3 (0.8)	3.6 (0.9)	3.7 (1.0)	3.9 (0.7)	4.0 (0.9)
Self-efficacy for self-monitoring dietary intake[e]	--	62.3 (25.7)	--	--	80.8 (22.7)	--
Motivation (TSRQ)						
amotivation	2.2 (1.3)	2.1 (1.1)	--	2.0 (1.3)	1.7 (1.2)	--
controlled motivation	3.7 (1.4)	3.5 (1.4)	--	2.9 (1.3)	2.6 (0.9)	--
autonomous motivation	5.9 (1.1)	5.9 (1.2)	--	6.3 (1.1)	6.0 (1.2)	--
Self-regulation for eating (TFEQ-R18)						
cognitive restraint	42.5 (10.3)	54.3 (13.0)	56.3 (13.2)	43.4 (14.8)	55.0 (13.7)	57.9 (18.1)
uncontrolled eating	50.8 (18.4)	45.3 (21.0)	39.6 (21.8)	38.3 (17.6)	38.0 (19.2)	30.0 (14.1)
emotional eating	57.5 (25.5)	51.0 (23.9)	47.9 (22.1)	44.4 (31.6)	42.7 (27.0)	32.2 (27.7)
Perceived stress (PSS-10)	16.8 (7.0)	18.7 (9.1)	18.4 (7.6)	10.3 (6.1)	14.1 (7.9)	10.7 (6.3)
Outcome expectations	99.7 (28.9)	92.2 (30.9)	--	101.3 (28.7)	99.4 (28.0)	--
Outcome realizations	--	--	81.6 (28.4)	--	--	88.1 (33.4)

Abbreviations: GLTEQ, Godin Leisure-Time Exercise Questionnaire; kcal, kilocalorie; MVPA, moderate-to-vigorous physical activity; PSS-10, the Perceived Stress Scale, 10 items; TFEQ-R18, the Three Factor Eating Questionnaire Revised 18 items; TSRQ, the Treatment Self-Regulation Questionnaire; WEL-SF, the Weight Efficacy Lifestyle Questionnaire Short-Form

[a] A completer's analysis is presented for these pilot study data. Unless otherwise noted, at baseline, data are collected from n=38/38 participants; at 1 month, data are collected from n=36/38 participants (Detailed arm: 17/18; Simplified arm: 19/20); at 3 months, data are collected from 35/38 participants (Detailed arm: 16/18; Simplified arm: 19/20).
[b] Caloric intake was assessed via the ASA24 dietary recall. There was 100% completion at baseline. N at 3 months: Detailed n=12/18; Simplified n=17/20.
[c] –, indicates not assessed at that particular time point.
[d] Step count was collected via the Fitbit Inspire 2 activity monitor. A one-week average is represented at each time point (baseline: 1st week of the intervention; 1 month: 5th week of the intervention; 3 months: 12 week of the intervention). N at each time point: baseline: Detailed arm: n=15/18; Simplified arm: n=20/20; 1 month: Detailed: n=15/18; Simplified: n=19/20. 3 month: Detailed: n=11/18; Simplified: n=17/20.
[e] The Detailed arm was asked about self-efficacy for tracking their foods on a daily basis whereas the Simplified arm was asked about self-efficacy for self-monitoring Red Zone Foods on a daily basis.
